# Oocyte Spontaneous Activation: An Overlooked Cellular Event That Impairs Female Fertility in Mammals

**DOI:** 10.3389/fcell.2021.648057

**Published:** 2021-03-08

**Authors:** Wei Cui

**Affiliations:** Department of Veterinary and Animal Sciences, Animal Models Core Facility, Institute for Applied Life Sciences (IALS), University of Massachusetts Amherst, Amherst, MA, United States

**Keywords:** meiosis, cell cycle, assisted reproduction, ovarian teratoma, multiple pronuclei, aneuploidy, metaphase arrest, triploid pronuclei

## Abstract

In mammals, including humans, mature oocytes are ovulated into the oviduct for fertilization. Normally, these oocytes are arrested at metaphase of the second meiosis (MII), and this arrest can be maintained for a certain period, which is essential for fertilization *in vivo* and oocyte manipulations *in vitro*, such as assisted reproduction in clinics and nuclear/spindle transfer in laboratories. However, in some species and under certain circumstances, exit from MII occurs spontaneously without any obvious stimulation or morphological signs, which is so-called oocyte spontaneous activation (OSA). This mini-review summarizes two types of OSA. In the first type (e.g., most rat strains), oocytes can maintain MII arrest *in vivo*, but once removed out, oocytes undergo OSA with sister chromatids separated and eventually scattered in the cytoplasm. Because the stimulation is minimal (oocyte collection itself), this OSA is incomplete and cannot force oocytes into interphase. Notably, once re-activated by sperm or chemicals, those scattered chromatids will form multiple pronuclei (MPN), which may recapitulate certain MPN and aneuploidy cases observed in fertility clinics. The second type of OSA occurs in ovarian oocytes (e.g., certain mouse strains and dromedary camel). Without ovulation or fertilization, these OSA-oocytes can initiate intrafollicular development, but these parthenotes cannot develop to term due to aberrant genomic imprinting. Instead, they either degrade or give rise to ovarian teratomas, which have also been reported in female patients. Last but not the least, genetic models displaying OSA phenotypes and the lessons we can learn from animal OSA for human reproduction are also discussed.

## Introduction

Except some species (e.g., canine), mammalian females ovulate mature metaphase-II (MII) oocytes into the oviduct following luteinizing hormone (LH)-triggered oocyte maturation and follicular rupture (Cui and Kim, 2007; Duan and Sun, 2019). Normally, these ovulated oocytes can maintain MII arrest for a certain period until fertilization occurs (Fissore et al., 2002; Yin et al., 2008). Maintaining at MII stage is essential for not only fertilization *in vivo* but also oocyte manipulations *in vitro*, such as assisted reproduction, nuclear transfer cloning, and other therapeutic approaches (Sun et al., 2014; Herbert and Turnbull, 2018; Matoba and Zhang, 2018). However, in some species and under certain circumstances, exit from MII occurs spontaneously without any obvious stimulation or morphological signs, which is so-called oocyte spontaneous activation (OSA). In this mini-review, we highlight insights gained on two types of OSA through various animal models and discuss the effects of OSA on human fertility and reproductive health.

## First Type of OSA

**FIGURE 1:**
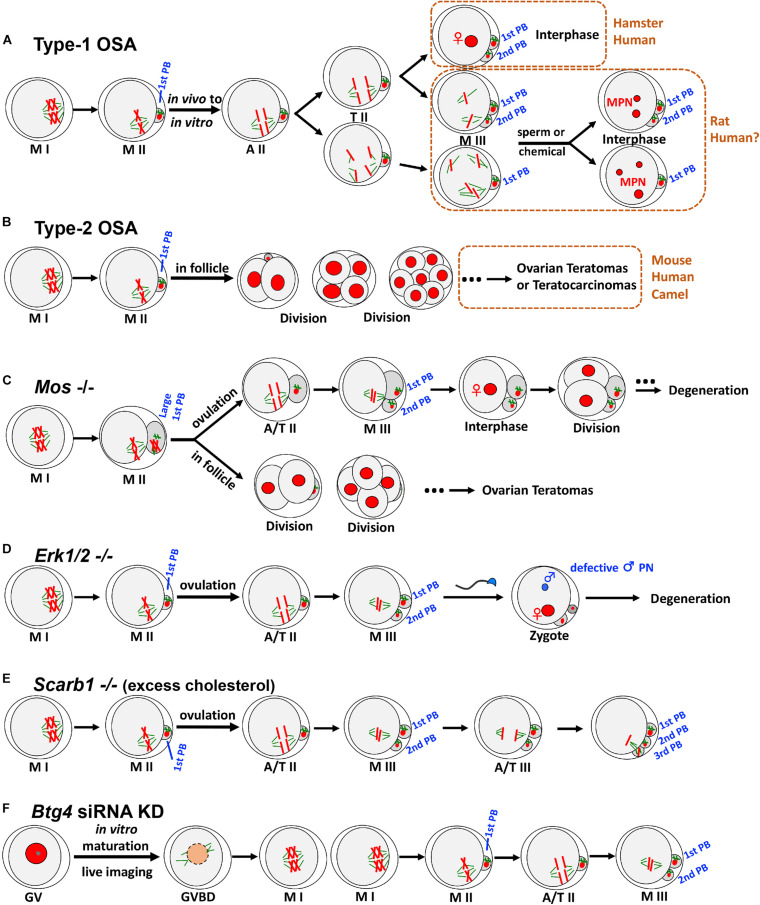
Two types of OSA and representative animal models exhibiting OSA phenotypes. **(A)** In type-1 OSA, ovulated mature oocytes can maintain MII arrest *in vivo*; however, once collected out without any obvious or artificial stimulation, oocytes undergo OSA. In hamster and some human IVF cases, OSA-oocytes can extrude 2nd PB and reach interphase with visible pronucleus. In rat, OSA-oocytes either extrude 2nd PB or not, depending on the oocyte postovulatory age, rat strain, external environment, and microtubule integrity. When OSA is finished, rat OSA-oocytes reach M-III arrest, with chromatids scattered around but no pronucleus formation. Once re-activated, those scattered chromatids will form multiple pronuclei (MPN), which may recapitulate certain MPN and aneuploidy cases observed in human fertility clinics. Notably, due to the nature of IVF lab protocol, only the consequence (3PN or MPN) has been reported, but information about the process prior to MPN formation was not available. We speculate that certain cases of human 3PN and MPN, especially after ICSI, are due to OSA. **(B)** In certain mouse strains, such as LT/Sv, some oocytes that have completed the first meiotic division can undergo type-2 OSA. Intrafollicular development of these parthenotes can cause ovarian teratomas (occasionally to teratocarcinomas). Similar phenotypes have also been reported in human and dromedary camel. **(C)** *Mos*– oocytes frequently produce large 1st PBs due to the failure of metaphase spindle movement. Ovulated MII oocytes undergo OSA to extrude 2nd PB and reach M-III, followed by pronucleus formation and cell divisions. Unovulated OSA-oocytes can initiate intrafollicular development to form ovarian teratomas. **(D)** Soon after ovulation, *Erk1/2*– oocytes undergo OSA and exit MII arrest with 2nd PB extruded spontaneously, reaching M-III stage but not into interphase. After fertilization, male pronucleus formation shows severe defects. **(E)** *Scarb1* knockout causes excess cholesterol deposition in oocytes, which does not affect oocyte maturation significantly. However, excess cholesterol in MII oocytes can induce an elevation of (Ca^2+^)i, leading to OSA and extrusion of 2nd PB to M-III after ovulation. Furthermore, this cholesterol-induced OSA can result in a further round of meiosis with extrusion of 3rd PB. **(F)** *Btg4* knockdown immature GV oocytes under live imaging can resume meiosis and reach MII. BTG4 deficiency causes a global delay in maternal mRNA degradation, and excess polyadenylated mRNA would occupy the translational machinery, which then leads to an insufficient capacity of the oocyte to translate the mRNAs that are essential for MII arrest, leading to OSA and reaching M-III. MI, metaphase of the first meiosis; MII, metaphase of the second meiosis; A/T, anaphase/telophase; PB, polar body; GV, germinal vesicle; GVBD, GV breakdown; KD, knockdown. For clarity, only four of 40 chromatids at MII are illustrated.

In the first type of OSA (type-1 OSA) (Figure 1A), ovulated mature oocytes can maintain MII arrest *in vivo* (in the oviduct); however, once collected out without any obvious or artificial stimulation, oocytes undergo OSA. In other words, these oocytes have very limited ability to maintain the MII arrest, and only oocyte recovery procedure itself can trigger parthenogenetic activation in these oocytes. This type of OSA has been known and studied in multiple species, mainly on rat (Keefer and Schuetz, 1982; Zernicka-Goetz, 1991) and golden hamster (Goud et al., 1998; Sun et al., 2002), together with case reports from human *in vitro* fertilization (IVF) clinics (Van Blerkom et al., 1994; Osman et al., 2019; Ye et al., 2020).

Morphological and cytoskeletal changes during type-1 OSA have been relatively well studied in rats. The initial separation of sister chromatids is similar to normal MII-to-AII (anaphase II) transition as seen in sperm or chemical-induced meiotic resumption and oocyte activation (Ross et al., 2006; Cui et al., 2012). Following this, oocytes either extrude the second polar body (2nd PB) or just exhibit a protrusion without 2nd PB extrusion or even no obvious change at all, depending on the oocyte postovulatory age, rat strain, external environment, and microtubule integrity (Zernicka-Goetz, 1991; Ross et al., 2006; Chaube et al., 2007; Cui et al., 2012). When OSA is done (normally around 6 h post *in vitro* culture), scattered chromatids and surrounding microtubules form multiple small spindle-like structures, reaching a new metaphase-like arrest (Zernicka-Goetz et al., 1993; Tomioka et al., 2007; Cui et al., 2012). Because the stimulation is minimal (oocyte collection itself and the following *in vitro* culture), this OSA is incomplete/abortive and cannot force oocytes into interphase, and therefore, no pronucleus formation occurs after OSA. Instead, OSA-oocytes enter a so-called “metaphase III-like” (M-III) arrest (Zernicka-Goetz, 1991). Notably, these OSA-oocytes can be re-activated by sperm or chemicals, and once re-activated, those scattered chromatids will form multiple pronuclei (MPN), which may recapitulate certain MPN and aneuploidy cases observed in human fertility clinics (Van Blerkom et al., 1984; Dozortsev et al., 1998; Hayes et al., 2001; Dai et al., 2017; Grigoryan et al., 2019). Different from rat, some hamster and human OSA-oocytes can reach interphase with visible pronuclei (Longo, 1974; Van Blerkom et al., 1994; Sun et al., 2002; Jiang et al., 2015; Osman et al., 2019; Ye et al., 2020).

## Mechanism and Control of Type-1 OSA

Although no obvious or artificial stimulation is applied on OSA-oocytes, substantial subtle changes could happen during oocyte collection and *in vitro* culture. Among all factors, exposure to cold and prolonged retention in the oviduct after animal sacrifice can significantly increase rat OSA (Keefer and Schuetz, 1982; Zernicka-Goetz, 1991; Kito et al., 2010). Another widely recognized factor is postovulatory aging, which has been shown in rat (Ben-Yosef et al., 1995; Chaube et al., 2007; Cui et al., 2012), golden hamster (Sun et al., 2002; Jiang et al., 2015), and human (Santos et al., 2003) to facilitate OSA (Table 1). Same as other vertebrates, the two most critical kinases of cytostatic factor (CSF), maturation-promoting factor (MPF), and mitogen-activated protein kinase (MAPK), are also involved in OSA (Tiwari et al., 2018). Previous studies revealed that rat oocytes carry only 40% MPF kinase activity of that in mouse oocytes (Ito et al., 2005), which could explain the susceptibility of rat oocytes to OSA, although variation in MPF activity has been detected among different rat strains (Hirabayashi et al., 2003; Ross et al., 2006; Sterthaus et al., 2009). As a master regulator of microtubule organization and spindle assembly during oocyte meiosis, MAPK, the other pivotal CSF, has also been studied in rat OSA. Different from sperm or chemical-induced oocyte activation where high MAPK activity still lasts several hours after the stimulation (Fan and Sun, 2004), rat OSA exhibits a quick decrease in both Mos and MAPK kinase (MEK)/MAPK (Ito et al., 2007; Table 1), which could explain the reason underlying the disintegrated microtubules and failure of 2nd PB extrusion during OSA in some rat strains (Cui et al., 2012; Prasad et al., 2015). Regarding the mechanism of re-entering the so-called M-III arrest after OSA, a plausible scenario is that defects in attachment of kinetochores and/or spindle assembly caused by premature MAPK decline could activate the spindle assembly checkpoint (SAC) proteins, which then mobilize cyclin B protein and actuate MPF activity (Cui et al., 2012).

**TABLE 1:** Factors that are involved in type-1 OSA.

Factor	Effect and how to control	References
Temperature	Avoid temperature change during oocyte collection from oviduct to medium	Zernicka-Goetz, 1991; Kito et al., 2010
Time interval between animal euthanasia and oocyte collection	Shorten the time interval and do animal sacrifice and oocyte collection one by one instead of a batch	Keefer and Schuetz, 1982; Kito et al., 2010
Oocyte postovulatory age	Avoid oocyte postovulatory aging *in vivo*	Ben-Yosef et al., 1995; Sun et al., 2002; Santos et al., 2003
Initial MPF level	Rat oocytes carry only 40% MPF kinase activity of that in mouse oocytes	Ito et al., 2005
Timing of MAPK decline	Rat OSA exhibits a quick decrease in both Mos and MAPK kinase (MEK)/MAPK	Ito et al., 2007
Spindle assembly checkpoint (SAC)	Premature MAPK decline disintegrates microtubules and activates the SAC proteins, which then mobilize cyclin-B protein and bring oocytes to M-III	Cui et al., 2012
Ca^2+^ and calmodulin-dependent protein kinase II (CaMKII)	Ca^2+^ and CaMKII cascade contribute to MPF inactivation and premature MAPK decline. Ca^2+^-free medium, Ca^2+^ chelator, Ca^2+^ channel blockers, NCX1 activator, and CaMKII inhibitors have been applied, but these methods cannot fully block OSA or can cause obvious side effects	Hayes et al., 2001; Sun et al., 2002; Ito et al., 2006; Chaube et al., 2007; Ito et al., 2007; Yoo and Smith, 2007; Cui et al., 2013
Cyclin-B degradation during OSA	To inhibit cyclin-B degradation and MPF inactivation, proteasome inhibitor MG132 was widely applied, but caution should be exercised due to its profound side effects	Zhou et al., 2003; Ito et al., 2007; Popova et al., 2009; Mizumoto et al., 2010; Cui et al., 2013
Rat strains	Different rat strains exhibit different MPF activities and distinct susceptibility to OSA	Hirabayashi et al., 2003; Ross et al., 2006; Sterthaus et al., 2009
Other factors	Nitric oxide, cyclin-dependent kinase 1, ubiquitin-proteasome pathway, and reactive oxygen species.	Tan et al., 2005; Premkumar and Chaube, 2015; Prasad and Chaube, 2016; Prasad et al., 2016a,b; Premkumar and Chaube, 2016

Similar to sperm and chemical-induced mammalian oocyte activation (Miao and Williams, 2012; Parrington et al., 2019), Ca^2+^ and calmodulin-dependent protein kinase II (CaMKII) are also involved in OSA attributing to MPF inactivation and probably a premature decline in Mos/MEK/MAPK (Ito et al., 2006; Ito et al., 2007; Yoo and Smith, 2007; Table 1). It is noteworthy that increase of intracellular free Ca^2+^ [(Ca^2+^)i] during OSA is insufficient (Cui et al., 2012; Premkumar and Chaube, 2013) compared with the pattern caused by sperm or chemical, which could explain why OSA is incomplete and abortive that cannot force oocytes into interphase to form pronucleus. To block increase of (Ca^2+^)i and its cascade during OSA, Ca^2+^-free medium (Hayes et al., 2001; Sun et al., 2002; Premkumar and Chaube, 2013), Ca^2+^ chelator (Ito et al., 2007), multiple Ca^2+^ channel blockers (Chaube et al., 2007; Yoo and Smith, 2007), and CaMKII inhibitors (Ito et al., 2006; Yoo and Smith, 2007) have been applied (Table 1), but these methods cannot fully block OSA or can cause obvious side effects [reviewed in Chebotareva et al. (2011)]. A novel physiological method focusing on sodium/calcium exchanger-mediated Ca^2+^ efflux has been demonstrated effective to block OSA, but this method cannot override the stimulation caused by enucleation during somatic cell nuclear transfer (SCNT) (Cui et al., 2013). To inhibit cyclin-B degradation and MPF inactivation, proteasome inhibitor MG132 was also widely applied (Zhou et al., 2003; Ito et al., 2007; Popova et al., 2009; Sterthaus et al., 2009; Mizumoto et al., 2010; Cui et al., 2013), but caution should be exercised due to its profound side effects [reviewed in Chebotareva et al. (2011)]. Furthermore, other factors have also been evaluated to better elucidate type-1 OSA, such as intracellular nitric oxide (Premkumar and Chaube, 2015; Prasad and Chaube, 2016), cyclin-dependent kinase 1 (Prasad et al., 2016a,b), ubiquitin-proteasome pathway (Tan et al., 2005), and reactive oxygen species (Premkumar and Chaube, 2016). To summarize, although more factors and pathways involved in type-1 OSA are emerging (Table 1), the nature of trigger and the best way to prevent the onset of OSA are still unclear. With advancement in rat and hamster genome editing especially under CRISPR/Cas9 system (Meek et al., 2017; Li et al., 2018), we hope a clear picture of type-1 OSA could be achieved soon.

## “Confusing” Terms

Given the theme of this mini-review and the following content (type-2 OSA) to discuss, it seems helpful to clarify some “confusing” terms here.

### Spontaneous Meiotic Resumption

Once mammalian oocytes are separated from the antral follicles and cultured under appropriate conditions, they can resume meiosis spontaneously from the diplotene stage of the first meiotic prophase to MII, which is also called *spontaneous maturation* (Liang et al., 2007; Pan and Li, 2019).

### Spontaneous Ovulation

Most mammals including women display a continuous cycling of reproductive hormones with ovulation occurring at regular intervals, which is different from those induced ovulators (e.g., rabbits, cats, and camelids) that copulation is responsible for hormone regulation and ovulation (Ratto et al., 2019).

### Postovulatory Oocyte Aging vs. OSA

Normally, mature oocytes can maintain MII arrest for a certain period *in vivo* or *in vitro*. If fertilization does not occur, oocytes undergo postovulatory oocyte aging, and too “aged” oocytes may GRADUALLY exit MII arrest with some initiating OSA (Pickering et al., 1988; Xu et al., 1997; Gordo et al., 2002; Miao et al., 2009). In short, long-time postovulatory oocyte aging may facilitate OSA both *in vivo* and *in vitro*, but OSA can also occur in very “young and fresh” oocytes, and OSA process is relatively much faster and uncontrollable (discussed in section “First Type Of OSA”).

## Type-2 OSA and Mechanism

Although oocytes spend majority of their life in the ovary and follicles, to become an embryo, it has to be ovulated from the follicle into oviduct for fertilization with sperm. However, OSA-induced embryogenesis is an exception. The second type of OSA (Figure 1B) occurs in ovarian oocytes within the follicles and it can initiate intrafollicular development to a certain stage. For example, in LT/Sv mice, a substantial portion of oocytes that have completed the first meiotic division can undergo OSA (Eppig et al., 1977). Although these ovarian OSA-embryos resemble normal until the blastocyst or even primitive streak stage, later on, most of them become disorganized and form ovarian teratomas (Stevens and Varnum, 1974). Usually, these teratomas are benign, but occasionally, they grow progressively and are malignant, containing multiple types of tissue and proliferating pluripotent stem cells (embryonal carcinoma cells that are called teratocarcinomas) (Stevens, 1980). Notably, these phenotypes were also reported in humans, including ovarian zygotes (Combelles et al., 2011), two-cell embryo (Padilla et al., 1987), four-cell embryo (Oliveira et al., 2004), and teratomas (Linder et al., 1975). To clarify, the existence of nuclei had been confirmed in all blastomeres of the above-mentioned human ovarian OSA-embryos, ruling out the possibly of cytoplasmic fragmentation, which is a relatively common phenomenon in aged unfertilized oocytes or during human preimplantation development (Alikani et al., 1999; Lord and Aitken, 2013).

In addition to spontaneous ovulators, type-2 OSA and ovarian teratoma have also been detected in induced ovulators, such as dromedary camel (*Camelus dromedarius*) (Mesbah et al., 2002, 2004). Similarly, OSA-oocytes can initiate intrafollicular development to blastocyst stage with clear inner cell mass and trophectoderm (Abdoon et al., 2007, 2020), suggesting the occurrence of the first cell lineage specification in these parthenotes (Cui and Mager, 2018; Ho et al., 2019). Although the underlying molecular mechanism that causes type-2 OSA is not fully understood yet, current knowledge from mouse models suggests that type-2 OSA and teratoma formation are multigenic traits (Eppig et al., 1996), involving genetic background (Lee et al., 1997; Ciemerych and Kubiak, 1998; Cheng et al., 2012; Abdoon et al., 2020), cytoskeletal arrangement and SAC (Albertini and Eppig, 1995; Maciejewska et al., 2009), companion somatic cells (Eppig et al., 2000), AMPK signaling (Downs et al., 2010; Ya and Downs, 2013), and hormonal regulation (Speirs and Kaufman, 1988).

## Genetic Models Displaying OSA Phenotypes

With success in embryonic stem cell (ESC)-mediated gene targeting and CRISPR/Cas9-mediated genome editing, more engineered animal models have been generated for studying mammalian oocyte meiosis. Here, we briefly review some examples.

*Mos* knockout female mice can produce MII oocytes; however, these oocytes cannot arrest at MII stage (Figure 1C). For those ovulated MII oocytes, they will spontaneously extrude 2nd PB and reach M-III, followed by pronucleus formation and cell divisions. Meanwhile, unovulated OSA-oocytes can initiate intrafollicular development, which then causes ovarian teratomas (Colledge et al., 1994; Hashimoto et al., 1994; Araki et al., 1996). In addition, *Mos*– oocytes frequently produce large first polar bodies (1st PBs) due to the failure of metaphase spindle movement (Choi et al., 1996; Verlhac et al., 1996). Given the phenotypes detected from the knockout mice, these models are valuable for studying human ovarian pathology and teratogenesis.

Although MOS/MEK/ERK cascade has been relatively well studied in oocyte meiosis, the explicit role of extracellular signal-regulated kinase (ERK) *in vivo* was not clear. Through the knockout of both *Erk1* and *Erk2* in mouse oocytes (Figure 1D), data indicates that *Erk1/2*– oocytes exit MII arrest and extrude 2nd PB spontaneously, reaching M-III stage. Different from *Mos*– oocytes, *Erk1/2*– MII oocytes do not exhibit large 1st PBs, and subsequent M-III oocytes display low frequency of pronucleus formation, explaining why ovarian teratomas were not detected in the females. Interestingly, ERK1/2 deletion also severely prevents male pronucleus formation after fertilization (Zhang et al., 2015), representing another major contributing cause of female infertility.

Female fertility can be affected by many factors, including diet and nutrient metabolism. Two recent studies using genetically modified mice revealed that maintenance of cholesterol within a physiological range during oocyte development and maturation is essential for female fertility. Excess cholesterol deposition in MII oocytes can induce an elevation of (Ca^2+^)i, which then triggers reduction in both MPF and MAPK, leading to OSA and extrusion of 2nd PB to M-III (Figure 1E). Different from all the above-mentioned OSAs, this cholesterol-induced OSA can result in multiple cell cycles, including execution of the third meiosis with extrusion of 3rd PB (Yesilaltay et al., 2014; Quiroz et al., 2020), which was found in partially activated oocytes (Kubiak, 1989). Importantly, this excess-cholesterol-induced OSA can be reversed both *in vivo* and *in vitro* (Yesilaltay et al., 2014; Quiroz et al., 2020), highlighting the possibility that cholesterol metabolism may underlie some woman infertility of unknown etiology and this could be cured with appropriate treatments.

In mammals, oocyte meiotic maturation not only produces a haploid gamete but also initiates maternal mRNA transition from stable to unstable (Wu and Dean, 2016; Sha et al., 2019), serving as a prolog to maternal-zygotic transition (MZT) which involves maternal mRNA destabilization and degradation. Recently, three laboratories independently identified BTG4 as a key mediator that links mRNA decay machinery and meiotic cell cycle progression, and loss of BTG4 causes a global delay in maternal mRNA degradation (Liu et al., 2016; Pasternak et al., 2016; Yu et al., 2016). In addition, BTG4 was also identified as essential for MII arrest (Figure 1F), because excess polyadenylated mRNA caused by *Btg4* knockdown could occupy the translational machinery, which then leads to an insufficient capacity of the oocyte to translate the mRNAs that are essential for MII arrest (e.g., mRNAs encoding EMI2), and all of this finally resulted in OSA to M-III (Pasternak et al., 2016). Interestingly, this OSA phenotype was not detected in knockout models, which could be due to the environment (*in vitro* live imaging vs. *in vivo*) and/or methodologies (difference in genetic compensation and specificity between knockdown and knockout).

## Discussion

We reviewed two types of OSA and representative animal models exhibiting OSA phenotypes due to genetic defects. Regarding type-1 OSA (*in vivo* to *in vitro*), we propose that more caution should be exercised during assisted human reproduction, as many steps could trigger OSA, such as oocyte retrieval (Muechler et al., 1989), cryopreservation (Gook et al., 1995), and intracytoplasmic sperm injection (ICSI) (Sultan et al., 1995). Furthermore, as learned from rat OSA, certain OSA-oocytes could show minimal morphological signs (e.g., sister chromatids separated or even scattered in cytoplasm but without 2nd PB or pronucleus formation). Therefore, we propose OSA should be considered for those unexplained abnormal fertilization with repeated triploid pronuclei (3PN) (Grigoryan et al., 2019) or even more pronuclei (e.g., up to 8PN) (Dai et al., 2017) after ICSI. Other lessons we can learn from animal models and issues that should be addressed are as follows: (1) time interval between oocyte pickup and IVF/ICSI. Currently, there is no consensus among clinics about this interval, and 2–6 h are widely accepted for a better cytoplasmic maturity but without aging (Van de Velde et al., 1998; Garor et al., 2015). This routine interval seems fine for most patients; however, for those that cannot achieve pregnancy after multiple cycles and especially with repeated 3PN or MPN, this interval probably needs to be avoided as OSA could be the reason that is much faster than natural aging. (2) Make everything ready for a rapid ICSI. For oocytes that are susceptible to OSA (Dozortsev et al., 1998; Morishita et al., 2019), all things should be well prepared before oocyte retrieval; right after which, a careful and rapid oocyte denudation and ICSI should follow to mitigate OSA-induced abnormal fertilization and possible aneuploidy.

A substantial number of patients cannot achieve successful pregnancy after multiple IVF cycles, and it is generally believed that genetic defects underlie many of these unrecognized pathologies (Conti and Franciosi, 2018; Cui, 2020). Dissecting the association between genetic variants and human OSA is challenging because the etiology is highly heterogeneous and patients have different genetic predispositions and epigenetic modifications (Marshall and Rivera, 2018; Ou et al., 2019), ages and lifestyles (Qiao et al., 2014; Grondahl et al., 2017), and exposures to diverse environments and pollutants (Peretz et al., 2014). To gain a better understanding of human oocyte meiosis and idiopathic infertility, animal models have been generated to define key factors and pathways involved in meiotic cell cycle regulation. Although more than 400 mutant mouse models with reproductive phenotypes have been established (Matzuk and Lamb, 2008), many genes and pathways regulating oocyte meiosis and OSA are still not fully delineated due to insufficient models and possible limitations when translating the information from mice to humans. With more mouse models being generated by the Knockout Mouse Program and the International Mouse Phenotyping Consortium^1^ and recent application of CRISPR/Cas9 in other species that can bypass barriers of ESCs and SCNT, we believe more essential genes will be screened out and more appropriate animal models (e.g., point mutation by knock-in) will be generated. We hope, with more precise animal models available, more sophisticated clinical protocols (Sachs et al., 2000; Socolov et al., 2015), faster genetic tests in clinics, more advanced assisted reproductive technologies (Smith and Takayama, 2017; Belli et al., 2019; Hawkins et al., 2021), and genetic diagnosis in preimplantation embryos, we will fully understand the underpinnings of human OSA, an overlooked meiotic instability problem that requires global attention (Premkumar et al., 2020).

## Author Contributions

WC conceived the study, prepared the figures, and wrote the manuscript.

## Conflict of Interest

The author declares that the research was conducted in the absence of any commercial or financial relationships that could be construed as a potential conflict of interest.

## Footnotes

1 https://www.mousephenotype.org/about-impc/about-komp/
